# Non-invasive Diagnosis and Prognosis Values of 3D Pseudocontinuous Arterial Spin Labeling and Optical Coherence Tomography Angiography in Proliferative Diabetic Retinopathy

**DOI:** 10.3389/fmed.2021.682708

**Published:** 2021-06-04

**Authors:** Yanli Hou, Shuai Song, Jiao Sun, Huihui Wang, Yanling Wang, Zhenchang Wang, Jing Li, Hongyang Li

**Affiliations:** ^1^Department of Ophthalmology, Beijing Friendship Hospital, Capital Medical University, Beijing, China; ^2^Deparment of Thoracic Surgery, Beijing Friendship Hospital, Capital Medical University, Beijing, China; ^3^Department of Radiology, Beijing Friendship Hospital, Capital Medical University, Beijing, China

**Keywords:** 3D-pcASL, OCTA, diabetic retinopathy, blood flow, optic nerve

## Abstract

**Background:** 3D Pseudocontinuous Arterial Spin Labeling (3D-PCASL) MRI and optical coherence tomography angiography (OCTA) have been applied to detect ocular blood flow (BF). We aim to characterize the ocular BF in diabetic retinopathy (DR) using 3D-PCASL and OCTA, to discuss the relationship between ocular and cerebral BF, and to evaluate their potential utility to assess the severity of DR.

**Methods:** A total of 66 participants (132 eyes) were included. Seventy-two eyes were classified in the proliferative diabetic retinopathy (PDR) group, and 60 were in the non-proliferative diabetic retinopathy NPDR group. Ocular and cerebral BF values were detected by 3D-PCASL using a 3.0T MRI scanner with two post-labeling delays (PLDs). Vessel density (VD)/perfusion density (PD) of the macular or peripapillary area were detected by OCTA. Parameters and clinical characteristics were compared between the PDR and NPDR eyes utilizing two-sample *t*-tests and chi-square tests. Spearman's rank correlation analysis, logistic regression analysis, and receiver operating characteristic curves (ROC) analyses were performed to evaluate the factors' role in DR severity.

**Results:** The perfusions of the retinal/choroidal plexus (RCP), optic nerve head (ONH)/optic nerve (ON), and VD/PD of macular/peripapillary area in the PDR group were significantly lower compared to the NPDR group (*p* < 0.05). They were protective factors for PDR [ORs = 0.842 for RCP (1.5 s PLD), 0.910 for ONH (1.5 s PLD), 0.905 for ON (both 1.5 and 2.5 s PLD), 0.707 for macular VD, 0.652 for peripapillary VD, *p* < 0.05, respectively]. Ocular BF had a positive correlation with BF of the occipital lobe (OL) and temporal lobe (TL) in the cerebrum. The BF of RCP (lower than 7.825 mL/min/100 g at 1.5 s PLD) indicated PDR [areas under the curve (AUCs) = 0.682, 95% CI: 0.588–0.777, sensitivity: 70.7% specificity: 63.9%]. The AUC of RCP (PLD = 1.5 s) BF combined with peripapillary VD was 0.841 (95% CI: 0.588–0.777, sensitivity: 75.9% specificity: 82.9%).

**Conclusions:** 3D-pcASL and OCTA may be effective non-invasive methods to measure ocular blood flow in DR patients and assess the severity of DR.

## Introduction

Diabetic retinopathy (DR) is an ocular manifestation that affects one-third of diabetes patients (1). It is also the leading cause of visual impairment and new-onset blindness in working-age adults (2). DR is characterized by microvascular dysfunction (3, 4), and, if left untreated, can result in retinal neovascularization, loss of vision, and pain. More than 90% of blindness cases caused by DR, however, can be prevented by early detection and appropriate intervention (5).

At present, fluorescein angiography (FA) is the gold standard method used to evaluate the severity of DR—but, due to its invasive nature, depth limitations, ocular media restrictions, and the adverse effects of dyes (6), it is unsuitable for either early diagnosis of DR or long-term monitoring of the condition. Recent work suggests that imaging methods focusing on neural blood perfusion may have predictive value in evaluating DR. Prior evidence shows that ocular vascular changes [i.e., hypoperfusion of the retina /choroid plexus (RCP) (7)], rather than structural lesions, may underly the progression of DR (8–13). Retinal blood perfusion is controlled by neurons. In one study in a DR model, the ocular arteriole and capillary failed to dilate in coordination with metabolic demands when neurons were stimulated by lights (14). This lack of coordination could lead to a cycle of neuronal dysfunction and neurodegeneration, leading to vision loss and an increasingly pro-inflammatory environment which could ultimately promote the progression of DR (15). Thus, doppler, laser speckle, and angiography techniques have been used to measure ocular blood flow (16). However, quantifying images, particularly of the choroid and optic nerve, is still challenging using these methods.

Arterial spin labeling (ASL) perfusion MRI is a relatively new, versatile, and non-invasive imaging technique that uses magnetically labeled water in arterial blood as an endogenous tracer. Because of its highly layered structure, ASL-MRI is particularly applicable for measuring RCP perfusion (17). In a murine model of DR, Muir et al. (18) found that both retinal and choroid blood flow was successfully detected by ASL, with a larger view field and without depth limitation. Peng et al. (19) have also recently used the novel pCASL technique to acquire ocular blood flow images and parameters in humans with DR. Other ocular vasculature profiles in DR, such as vessel density (VD) and perfusion density (PD), can be detected by OCTA, an alternative non-invasive technique (20, 21).

Here, we use 3D-pCASL and OCTA to quantify RCP and ON blood perfusion changes throughout the progression of DR, and evaluate their potential utility to assess DR severity.

## Materials and Methods

### Subjects

Our prospective cross-sectional study was approved by the medical research ethics committee of Beijing Friendship Hospital at Capital Medical University. Written informed consent was obtained from all participants.

From November 2018 to December 2019, DR patients were recruited from the ophthalmology department of the Beijing Friendship Hospital at Capital Medical University. Exclusion criteria included: (1) type-1 diabetes mellitus; (2), Ocular vascular diseases, such as retinal vein occlusion; (3), a history of other retinal or optic nerve diseases, glaucoma, uveitis; (4), any systemic diseases that could have affected ocular blood flow, such as arteritis; and (5), contraindications for MRI.

Diagnosis of type 2 diabetes mellitus (DM) in each patient was confirmed by an internal medicine consultant. DR was confirmed by an ophthalmologist using fundus imaging and fluorescence fundus angiography (FFA). All recruited patients underwent detailed ophthalmological investigations, including best-corrected visual acuity (BCVA)–recorded as the logarithm of the minimum angle of resolution (logMAR)– slit-lamp biomicroscopy, intraocular pressure measurements, and fundus color photography (Kowa Nonmyd WX; Kowa Company Ltd., Japan) after full mydriasis and FFA. FFA images were read by a retina-trained specialist.

Images of eyes were categorized into a PDR group and an NPDR group, according to the International Clinical Diabetic Retinopathy Severity Scale (4). The PDR group was further categorized into two subgroups, according to BCVA: group A (logMAR ≥0.3) and group B (logMAR < 0.3).

### MRI Data Acquisition

MR imaging was carried out using a 3.0 T Ingenia scanner (Philips Healthcare, Best, Netherlands) equipped with a commercial body coil for transmission and a 16-channel head coil for reception. Conventional T2-weighted fast-spin-echo and 3D time-of-flight (TOF) MR angiography images were obtained before the ASL sequence to exclude patients with brain lesions or arterial stenosis. All patients were required to keep their eyes closed and stay motionless during the examinations.

Blood flow (BF) was imaged utilizing the pcASL technique. The gradients adopted an unbalanced scheme and the labeling parameters were optimized to decrease sensitivity to the off-resonance effect [post-labeling delay, PLD, = 1.5 seconds; (repetition time, TR = 3,814 ms; echo time, TE = 13 ms; bandwidth, BW = 7763.2 Hz); PLD = 2.5 s (TR = 4.928 ms; TE = 12 ms; BW = 8134.4 Hz); flip angle = 90; label distance = 90 mm]. Images were acquired using the multishot TSE sequence (slice thickness = 3.5 mm, number of slices = 20, slice orientation = transverse, slice gap = 0, field of view = 240 × 240 mm, in-plane resolution = 3 × 3 mm, matrix = 64 × 60, reconstruction matrix = 80, and number of excitations = 2).

### ASL Data Quantification

The RCP blood flow map was automatically derived from the ASL images using a dedicated workstation (IntelliSpace Portal Release v. 7.0.4.20175, Philips). A region of interest (ROI) in the RCP map was placed independently by an experienced radiologist and an experienced ophthalmologist at the level of the optic nerve. The two specialists reached an agreement on how to draw ROIs, which were two-dimensional and covered as much of the RCP as possible. The average value of each ROI was calculated automatically and then analyzed.

### Optical Coherence Tomography Angiography (OCTA) Measurement

OCTA examination was completed using the 68 kHz Zeiss Cirrus HD-OCT 5000 with AngioPlex at a wavelength of 840 nm (Carl Zeiss Meditec, Dublin, CA, USA) (22). Volumetric scans were processed using optical microangiography (OMAG) algorithms to generate flow images. The OMAG algorithm analyzes differences in both phase and intensity information from repeated B-scans to quantify motion contrast. The superficial capillary plexus (SCP) was extended from the internal limiting membrane to the inner plexiform layer.

A macular and optic disc-centered 6 × 6 mm scan was taken to investigate microvasculature. Each 6 × 6 mm scan contained 350 A-scans in each B-scan, and each B-scan was repeated two times at the same location. The vessel density (VD) was defined as the total length of perfused vasculature per unit area in a region of measurement, and perfusion density (PD) was defined as the total area of perfused vasculature per unit area in a region of measurement. Cirrus OCTA software (ver. 10.0) automatically calculated the value of the SCP according to the Early Treatment of Diabetic Retinopathy Study (ETDRS) subfields. We analyzed the macular VD, macular PD, peripapillary VD, and peripapillary PD in SCP of the full field of view (FOV).

### Statistical Analysis

All statistical analyses were performed using SPSS statistical software (version 26.0, SPSS Inc., Chicago, Illinois, USA). Data were expressed as the mean ± standard deviation. Two-sample *t*-tests and chi-square tests were performed to compare differences between the NPDR and PDR groups. Spearman's correlation analyses were utilized to evaluate the correlation between BF in the RCP and cerebrum. Binary Logistic regression analysis was used to obtain odds ratios (ORs). Diagnostic accuracy of RCP blood flow in DR grades that were measured by 3D-pCASL and OCTA was evaluated using receiver operating characteristic (ROC) curves. *P-*values < 0.05 were considered statistically significant.

## Results

### Patient Demographic and Clinical Characteristics

A total of 66 DR patients (132 eyes) were included, with the PDR group consisting of 38 patients (72 eyes), and the NPDR group consisting of 32 patients (60 eyes). Four patients had one eye included in the PDR group and the other in the NPDR group. There were 45 eyes in group A and 22 eyes in group B. No significant differences were identified between two groups in BCVA, IOP, age, gender, or other clinical indexes (Table 1).

**Table 1:** Patient demographics and clinical characteristics.

	**Group PDR**	**Group NPDR**	***P***
	**(*n* = 72)**	**(*n* = 60)**	
Age (years)	60.01 ± 7.09	58.22 ± 8.33	0.183
Gender (Male/Female)	38/34	38/22	0.222
BCVA, logMAR	0.33 ± 0.45	0.24 ± 0.30	0.201
IOP (mmHg)	15.06 ± 3.54	15.69 ± 2.55	0.279
BMI	25.44 ± 2.28	25.30 ± 2.88	0.725
MABP	98.00 ± 8.47	99.55 ± 9.10	0.323
HBP (Yes/No)	50/22	36/24	0.257
CHD (Yes/No)	4/68	4/56	1.000
CI (Yes/No)	15/57	13/47	0.907
Smoking (Yes/No)	28/44	26/34	0.605
Drinking (Yes/No)	20/52	18/42	0.779

*BCVA, best-corrected visual acuity; IOP, intraocular pressure; BMI, body mass index; MABP, mean arterial blood pressure; HBP, high blood pressure; CHD, coronary heart disease; CI, cerebral infarction*.

### Comparison BF and VD/PD Values of RCP and ON

**Figure 1:**
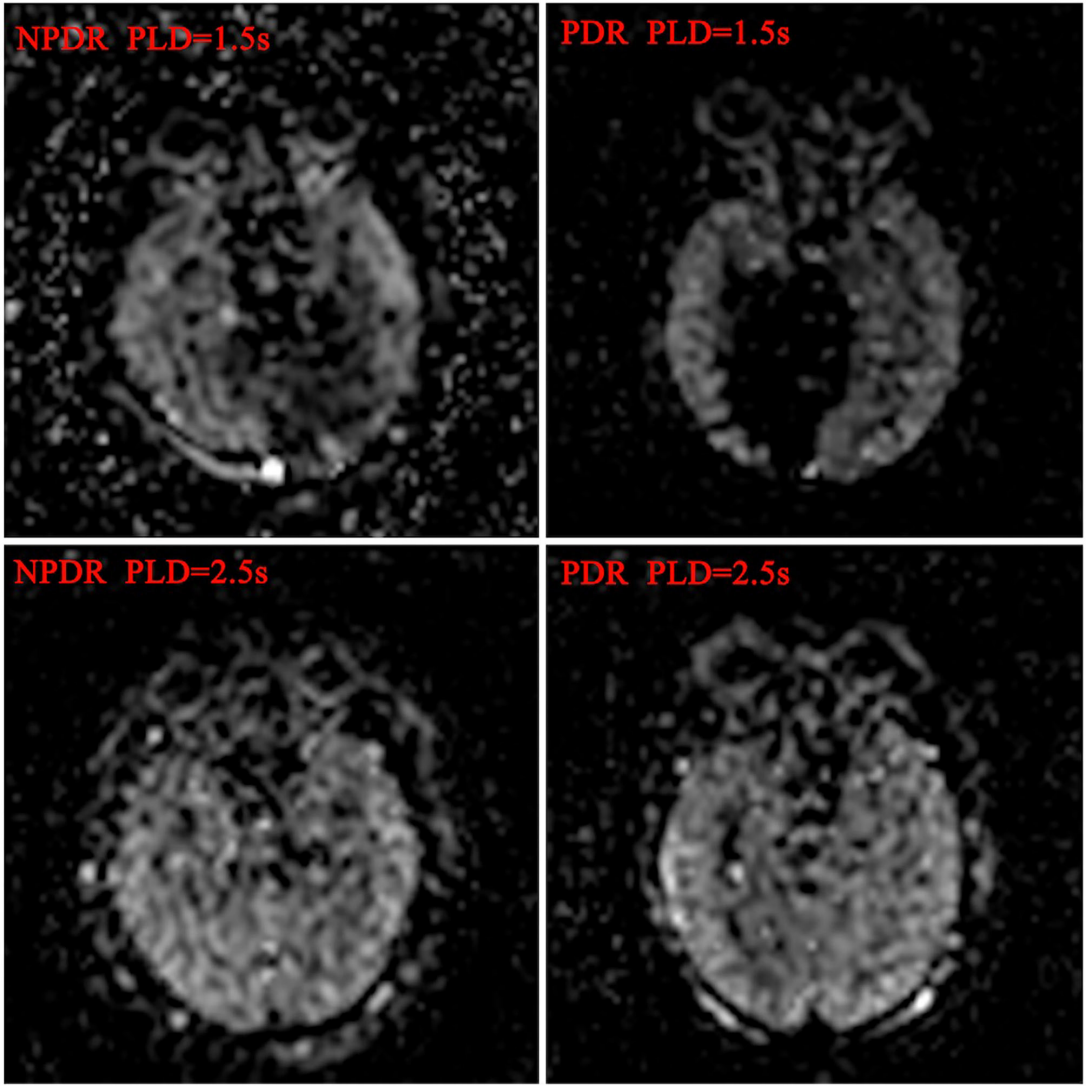
Blood flow maps of ocular and cerebral in DR patients. The ocular and cerebral blood flow maps was automatically derived from the ASL images at postlabeling delay 1.5 and 2.5 s, respectively.

The BF values of RCP and ONH in PDR group were significantly lower than the NPDR group at 1.5 s PLD, but not at 2.5 s PLD (RCP, 7.19 ± 3.30 vs. 9.93 ± 5.26 mL/min/100 g; ONH, 7.78 ± 4.38 vs. 10.23 ± 6.53 mL/min/100 g *p* < 0.05, respectively). The BF values of ON in the PDR group were notably lower than the NPDR group at 2 PLD (PLD = 1.5 s, 7.12 ± 3.18 vs. 9.24 ± 6.53 mL/min/100 g; PLD = 2.5 s, 10.43 ± 5.31 vs. 13.91 ± 6.86 mL/min/100 g *p* < 0.05, respectively). There were no differences between the BF values of OL and TL between the two groups at either PLDs (Figure 1). The BF value of ON in group A was 8.65 ± 4.38, significantly lower than group B [which was 11.86 ± 15.42 (*p* = 0.018) at 2.5 s PLD].

**Figure 2:**
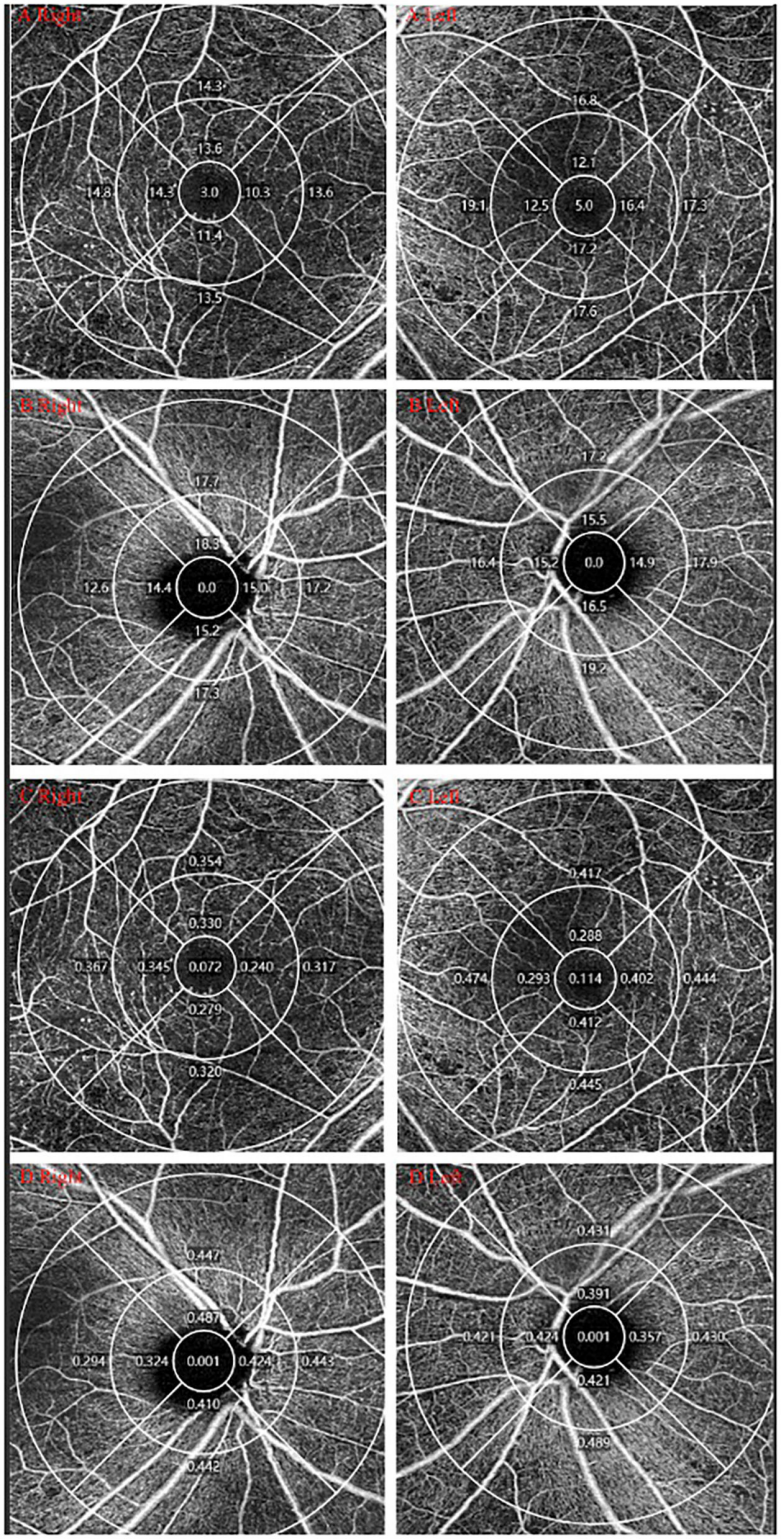
OCTA measurement of DR. One DM patients was diagnosed with PDR in the right eye and NPDR in the left eye. **(A,B)** VD of macular/peripapillary area in both eyes; **(C,D)** PD of macular/peripapillary area in both eyes.

The average VD and PD of the macular and peripapillary area in the PDR group were remarkably lower than in the NPDR group (*P* < 0.001 for both; see Table 2, Figure 2).

**Table 2:** BF/VD/PD values of the ocular and optic nerve.

**3D-PcASL**	**Field of view**	**Group PDR**	**Group NPDR**	***P***
		**(*n* = 74)**	**(*n* = 56)**	
BF (mL/min/100 g, PLD = 1.5 s)	RCP	7.19 ± 3.30	9.93 ± 5.26	<0.001^**^
	ONH	7.78 ± 4.38	10.23± 6.53	0.006^*^
	ON	7.12 ± 3.18	9.24 ± 6.53	0.011^*^
	TL	12.41 ± 7.28	13.78 ± 7.69	0.303
	OL	12.89 ± 9.84	11.18 ± 8.70	0.307
BF (mL/min/100 g, PLD = 2.5 s)	RCP	11.11 ± 5.31	12.13 ± 4.83	0.172
	ONH	11.80 ± 5.58	13.14 ± 5.16	0.115
	ON	10.43 ± 5.31	13.91 ± 6.86	0.001^*^
	TL	21.64 ± 9.99	22.47 ± 8.29	0.622
	OL	28.46 ± 11.25	30.43 ± 10.55	0.326
**OCTA**		**Group PDR**	**Group NPDR**	***P***
		**(*****n*** **=** **35)**	**(*****n*** **=** **40)**	
VD	Macular	12.37 ± 2.68	14.74 ± 2.54	<0.001^**^
	Peripapillary	14.38 ± 2.89	16.51 ± 1.67	<0.001^**^
PD	Macular	0.30 ± 0.069	0.36 ± 0.065	<0.001^**^
	Peripapillary	0.36 ± 0.084	0.42 ± 0.039	<0.001^**^

*BF, blood flow; RCP, retina /choroid plexus; ONH, optic nerve head; ON, optic nerve; TL, temporal lobe; OL, occipital lobe; VD, vessel density; PD, perfusion density*;

* *P < 0.05*;

** *P < 0.001*.

### The Relationship Between BF of Ocular and Cerebrum

Spearman correlation analysis was performed to explore the relationship between ocular and cerebral perfusion (Table 3). Spearman's correlation coefficients between BF of RCP/ONH/ON and TL at 1.5 s PLD were highly significant, with values of 0.580, 0.458, and 0.385 (*P* < 0.001 for all). Spearman's correlation coefficients between BF of RCP/ONH/ON and OL at 2.5 s PLD were also highly significant, with values of 0.239, 0.403, and 0.244 (*P* = 0.006, *P* < 0.001, *P* = 0.005, respectively).

**Table 3:** Spearman's rank correlation analysis between ocular and cerebral BF.

			**Correlation coeffificient**	***P-*value**
PLD = 1.5 s	TL	RCP	0.580^**^	< 0.001
		ONH	0.458^**^	< 0.001
		ON	0.385^**^	< 0.001
	OL	RCP	0.173^*^	0.048
		ONH	0.139	0.113
		ON	0.096	0.272
PLD = 2.5 s	TL	RCP	0.329^*^	0.000
		ONH	0.280^**^	0.001
		ON	0.147	0.095
	OL	RCP	0.239^**^	0.006
		ONH	0.403^**^	< 0.001
		ON	0.244^**^	0.005

*BF, blood flow; RCP, retina/choroid plexus; ONH, optic nerve head; ON, optic nerve; TL, temporal lobe; OL, occipital lobe; VD, vessel density; PD, perfusion density*;

* *P < 0.05*;

** *P < 0.001*.

### Logistic Regression Analysis of Factors Associated With PDR

Hierarchical data logistic regression was used to examine the association between BF of RCP/ONH/ON and VD/PD of macular/peripapillary area and DR degrees. Logistic regression analysis revealed that the BF of RCP/ONH/ON was a protective factor for PDR at 1.5 s PLD [ORs = 0.842 (95% CI: 0.764–0.842, *P* < 0.001), 0.910 (95% CI: 0.851–0.974, *P* < 0.05), 0.905 (95% CI: 0.839–0.976, *P* < 0.05)]. The BF of ON at 2.5 s PLD was also a protective factor for PDR. [OR = 0.905 (95% CI: 0.849–0.964, *P* < 0.05)]. VD/PD of the macular/peripapillary areas were all protective factors for PDR (Table 4).

**Table 4:** Logistic regression analysis of factors associated with PDR.

		**Unstandardized coefficients**		***P***	**OR**	**95% CI**	
		**B**	**Standard error**			**Lower bound**	**Upper bound**
BF	RCP(PLD = 1.5 s)	−0.172	0.049	0.000	0.842	0.764	0.927
	ONH(PLD = 1.5 s)	−0.094	0.035	0.006	0.910	0.851	0.974
	ON(PLD = 1.5 s)	−0.100	0.039	0.010	0.905	0.839	0.976
	ON(PLD = 2.5 s)	−0.100	0.032	0.002	0.905	0.849	0.964
VD	Macular	−0.346	0.104	0.001	0.707	0.577	0.867
	Peripapillary	−0.427	0.132	0.001	0.652	0.504	0.845
PD	Macular	−13.173	4.041	0.001	0.000	0.000	0.005
	Peripapillary	−15.732	4.788	0.001	0.000	0.000	0.002

*PDR, proliferative diabetic retinopathy; BF, blood flow; RCP, retina /choroid plexus; PLD, post labeling delays; B, regression correlation coefficient; OR, odds ratio; CI, confidence interval*.

### ROC Analysis

**Figure 3:**
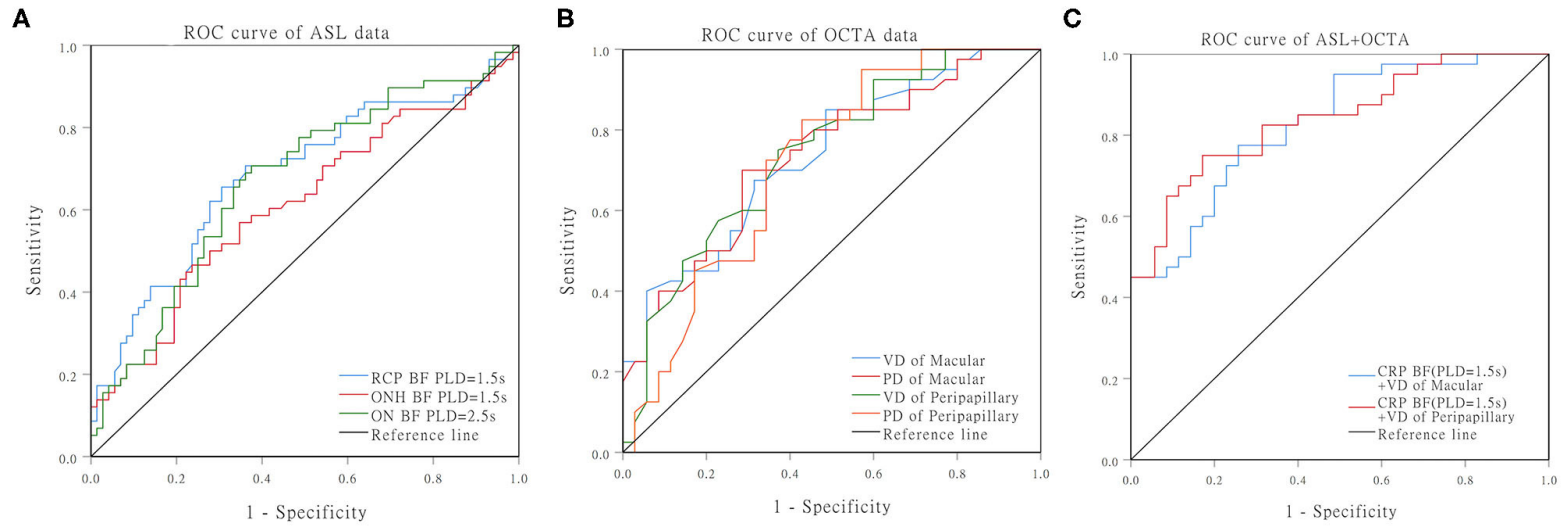
ROC curves for distinguishing PDR from NPDR. **(A)** ROC curves of the BF of RCP/ONH/ON that were measured by ASL. **(B)** ROC curves of the VD/PD of the macular/peripapillary areas that were measured by OCTA. **(C)** ROC curves of the RCP (PLD = 1.5 s) BF combined with peripapillary VD.

Finally, ROC curve analysis showed that, at 1.5 s PLD, the RCP BF cutoff value for the diagnosis of PDR was 7.825 mL/min/100 g, and AUC was 0.682 (95% CI: 0.588–0.777, sensitivity: 70.7% specificity: 63.9%). The ON BF cutoff values at 2.5 s PLD for the diagnosis of PDR was 10.54 mL/min/100 g, and AUC was 0.669 (95% CI: 0.575–0.763, sensitivity: 70.7% specificity: 62.5%). The VD cutoff values for the macular and peripapillary areas for the diagnosis of PDR were 12.45 and 15.70 [AUC: 0.734 (95% CI: 0.622–0.845, *P* < 0.001), AUC: 0.738 (95% CI: 0.625–0.850, *P* < 0.001), respectively]. The PD cutoff values for the macular and peripapillary areas for the diagnosis of PDR were 0.338 and 0.389 [AUC: 0.732 (95% CI: 0.619–0.844, *P* < 0.001) AUC: 0.716 (95% CI: 0.596–0.835, *P* < 0.001), respectively]. The AUC of combined BF for RCP (PLD = 1.5 s) and macular VD was 0.828 (95% CI: 0.738–0.918, *P* < 0.001 sensitivity: 77.5% specificity: 74.3%). The AUC of combined BF for RCP (PLD = 1.5 s) and peripapillary VD was 0.841 (95% CI: 0.753–0.928, *P* < 0.001, sensitivity: 75.9% specificity: 82.9%; Table 5, Figure 3).

**Table 5:** The AUC and cutoff values of BF, VD, and PD for the differential diagnosis of PDR.

		**AUC**	**95% CI**	**Cut-off point**	**Sensitivity**	**Specificity**
BF	RCP (PLD = 1.5 s)	0.682	0.588–0.777	7.825	0.707	0.639
	ONH(PLD = 1.5 s)	0.613	0.514–0.712	10.465	0.466	0.764
	ON(PLD = 2.5 s)	0.669	0.575–0.763	10.540	0.707	0.625
VD	Macular	0.734	0.622–0.845	12.450	0.850	0.514
	Peripapillary	0.738	0.625–0.850	15.700	0.750	0.629
PD	Macular	0.732	0.619–0.844	0.338	0.700	0.714
	Peripapillary	0.716	0.596–0.835	0.389	0.825	0.571
BF+VD	RCP(PLD = 1.5 s)+ Macular	0.828	0.738–0.918	0.557	0.775	0.743
	RCP(PLD = 1.5 s)+Peripapillary	0.841	0.753–0.928	0.515	0.759	0.829

*PDR, proliferative diabetic retinopathy; BF, blood flow; RCP, retina /choroid plexus; PLD, post labeling delays; AUC, area under the curve; CI, confidence interval*.

## Discussion

Here, we detected BF within the RCP, ON, and cerebrum in different stages of DR using multi-PLD 3D-pCASL, and measured the VD/PD of SCL in the macular and peripapillary areas using OCTA.

As DR progresses, we found that BF of the RCP and ONH significantly decreases at 1.5 s PLD, and BF of the ON decreases at both 1.5 and 2.5 s PLDs. We also found that BF of the ON in PDR patients with severe visual impairment was much lower at 2.5 s PLD. Hypoperfusion in the ocular and ON were risk factors for the development of PDR. Furthermore, the decreased BF of RCP/ONH/ON, and the decreased VD/PD of SCP in both the macular and peripapillary areas indicated more advantage stage of DR. BF of the RCP had the highest diagnostic efficiency at 1.5 s PLD, followed by ON at 2.5 s PLD in PDR patients. The two non-invasive methods combined had higher AUC, sensitivity, and specificity in diagnosing PDR than either method alone.

Interestingly, as DR progressed, both cerebral blood flow (CBF) and ocular blood flow decreased. The cerebrum may play a key role in vision formation and transmission. Previous studies (23, 24) have demonstrated that vision impairment causes reductions in cerebral BF, as well as functional changes, especially in vision-related areas such as the TL and occipital lobe (OL). However, other studies (25, 26) have found increased CBF in visually impaired patients. In our research, the RCP BF had a positive correlation with TL and OL BF at both PLDs. CBF had a decreasing trend with DR severity, but there was no statistical difference between PDR and NPDR groups. A possible explanation for this finding is that ocular adjustment and adaptation in DR can trigger a compensatory process that increases blood perfusion within the visual cortex.

One impact of DR is that normal retinal vascular autoregulation is disturbed. Vasoconstriction, provoked by protein kinase C or renin-angiotensin (27), ultimately leads to capillary occlusion and tissue hypoperfusion, or even non-perfusion. Neuronal dysfunction in DR may appear earlier than microvascular lesions, and DR may progress to advanced stages without producing any immediate symptoms. Thus, it is critical to determine appropriate intervention points, as well as to determine whether further invasive treatment methods would be able to prevent vision loss in DR patients. Current methods classify DR using fundus photography (28, 29) on the basis of the severity of certain clinical hallmarks (i.e., microaneurysms, capillary non-perfusion, leakage, and neovascularization). However, the fluorescein dye used in this process may lead to nausea, vomiting, itching, urticaria, and even anaphylaxis (6), and is unable to be used in vulnerable patients populations– including the elderly, children, patients with difficult venous access, and patients with renal insufficiency. Furthermore, FA detects only ~30% of the total retinal surface area (5). Other previous methods used to examine DR progression (30) include measuring ocular blood flow in different stages of DR by fundus photography, Doppler velocimetry, the blue field entoptic technique, and pneumotonometry. Using the Doppler device, Cuypers (8) and Nagaoka and Schocket et al. (7, 12) found that RCP BF was lower in PDR than in NPDR patients.

Other research groups have used ASL perfusion MRI to estimate CBF (31). With ASL, BF can be measured quantitatively in units of mL/min/100 g. The ASL technique is superior to existing perfusion imaging techniques because it is generally more sensitive to smaller vessels. It has a larger field of view with no depth limitations (32–34), meaning that BF in the back of the ocular field can be fully evaluated. Using ASL blood-flow MRI, Eric R (18) found that, in a mouse model of diabetes, reduced choroid and retinal ocular BF was an indicator of DR. Peng and Zhang (19, 34) were the first to investigate the feasibility of ASL to measure BF in the normal human retina. As a recently developed ASL technique, pCASL uses high sensitivity array coils, high SNR, and tagging efficiency to overcome the limitations of PASL and CASL. Further, 3D-pCASL is recommended in clinical studies (35, 36).

OCTA is another alternative non-invasive method that has been widely used in DR patients. It utilizes the motion of erythrocytes to detect BF in the retinal capillaries and potentially provides insights into retinal pathophysiology. Several researchers have detected microvascular details and capillary changes in DR patients (20, 21, 37–40). Hwang et al. (21) found VD and perifoveal PD were significantly reduced in the more advanced stages of DR (41, 42). Our study showed that VD and PD of both macular and peripapillary areas in PDR patients were reduced compared to NPDR patients.

Various forms of diabetic optic neuropathy can also accompany the progression of DR, and can eventually threaten vision (43). The optic nerve is impacted by impaired ocular microcirculation in DR. ONH perfusion can be measured by OCTA, but few techniques are available for the optic nerve because of its deep location. Here, we successfully quantified ON BF using a 3D-pCASL technique that has a large field of view without depth limitations. Perfusion of the ON was lower in PDR than NPDR eyes, and even worse in PDR eyes with low visual acuity.

## Conclusion

Our findings suggest that multi-PLD 3D-pcASL and OCTA offer non-invasive, replicable, and highly diagnostically efficient methods for tracking perfusion changes in the RCP and ON. They may also be reliable techniques to assist in categorizing DR stage and monitoring disease progression.

## Limitations

Our study utilized a retrospective design and was limited to a single center and absence of control group. Stringent inclusion criteria also limited our sample size to 132 eyes, which may have underpowered some aspects of the study. Due to the retrospective nature of the study, we could not achieve uniform ASL acquisition at a set time point. There could also have been a delay between symptoms and inclusion in the study. Additionally, an admission rate bias for patients in whom ASL was performed may have prevented us from having patients with comparable illness severity. The possibility of a measurement bias for imaging is also possible whenever manual measurements are used.

## Data Availability Statement

The original contributions presented in the study are included in the article/supplementary material, further inquiries can be directed to the corresponding authors.

## Ethics Statement

The studies involving human participants were reviewed and approved by This study was approved by the BFH Ethics Committee and was conducted following the latest iteration of the Declaration of Helsinki (version: 2019-P2-201-01). The patients/participants provided their written informed consent to participate in this study.

## Author Contributions

HL and JL was involved in the conception and design of the study. HW and ZW reviewed the orbit MRI images. YH and SS contributed to the acquisition, analysis and interpretation of data as well as drafting the manuscript and revising it critically. JS and YW were the duty of diagnosis the disease. All authors have provided final approval of the version to be published. All authors have agreed to be accountable for all aspects of the work in ensuring that questions related to the accuracy or integrity of any part of the work are appropriately investigated and resolved.

## Conflict of Interest

The authors declare that the research was conducted in the absence of any commercial or financial relationships that could be construed as a potential conflict of interest.

## References

[1] Yau JW Rogers SL Kawasaki R Lamoureux EL Kowalski JW Bek T. Global prevalence and major risk factors of diabetic retinopathy. Diabetes Care. (2012) 35:556–64. 10.2337/dc11-1909 22301125 PMC3322721

[2] Cheung N Mitchell P Wong TY. Diabetic retinopathy. Lancet. (2010) 376:124–36. 10.1016/S0140-6736(09)62124-3 20580421

[3] Mahajan N Arora P Sandhir R. Perturbed biochemical pathways and associated oxidative stress lead to vascular dysfunctions in diabetic retinopathy. Oxidative Med Cell Longevity. (2019) 2019:8458472. 10.1155/2019/8458472 30962865 PMC6431380

[4] Wilkinson CP Ferris FL III Klein RE Lee PP Agardh CD Davis M. Proposed international clinical diabetic retinopathy and diabetic macular edema disease severity scales. Ophthalmology. (2003) 110:1677–82. 10.1016/S0161-6420(03)00475-5 13129861

[5] Sadda SR Nittala MG Taweebanjongsin W Verma A Velaga SB Alagorie AR. Quantitative assessment of the severity of diabetic retinopathy. Am J Ophthalmol. (2020) 218:342–52. 10.1016/j.ajo.2020.05.021 32446737

[6] Kornblau IS El-Annan JF. Adverse reactions to fluorescein angiography: a comprehensive review of the literature. Surv Ophthalmol. (2019) 64:679–93. 10.1016/j.survophthal.2019.02.004 30772364

[7] Nagaoka T Kitaya N Sugawara R Yokota H Mori F Hikichi T. Alteration of choroidal circulation in the foveal region in patients with type 2 diabetes. Br J Ophthalmol. (2004) 88:1060–3. 10.1136/bjo.2003.035345 15258025 PMC1772269

[8] Cuypers MH Kasanardjo JS Polak BC. Retinal blood flow changes in diabetic retinopathy measured with the Heidelberg scanning laser Doppler flowmeter. Graefes Arch Clin Exp Ophthalmol. (2000) 238:935–41. 10.1007/s004170000207 11196354

[9] Kraśnicki P Mariak Z Ustymowicz A Proniewska-Skretek E. Assessment of blood flow in the ocular circulation in type 2 diabetes patients with Color Doppler imaging. Klin Oczna. (2006) 108:294–8. 17290827

[10] Savage HI Hendrix JW Peterson DC Young H Wilkinson CP. Differences in pulsatile ocular blood flow among three classifications of diabetic retinopathy. Invest Ophthalmol Vis Sci. (2004) 45:4504–9. 10.1167/iovs.04-0077 15557461

[11] MacKinnon JR McKillop G O'Brien C Swa K Butt Z Nelson P. Colour doppler imaging of the ocular circulation in diabetic retinopathy. Acta Ophthalmol Scand. (2000) 78:386–9. 10.1034/j.1600-0420.2000.078004386.x 10990037

[12] Schocket LS Brucker AJ Niknam RM Grunwald JE DuPont J Brucker AJ. Foveolar choroidal hemodynamics in proliferative diabetic retinopathy. Int Ophthalmol. (2004) 25:89–94. 10.1023/B:INTE.0000031744.93778.60 15290887

[13] Movaffaghy A Chamot SR Dosso A Pournaras CJ Sommerhalder JR Riva CE. Effect of isometric exercise on choroidal blood flow in type I diabetic patients. Klin Monbl Augenheilkd. (2002) 219:299–301. 10.1055/s-2002-30665 12022023

[14] Kornfield TE Newman EA. Regulation of blood flow in the retinal trilaminar vascular network. J Neurosci. (2014) 34:11504–13. 10.1523/JNEUROSCI.1971-14.2014 25143628 PMC4138352

[15] Du Y Veenstra A Palczewski K Kern TS. Photoreceptor cells are major contributors to diabetes-induced oxidative stress and local inflammation in the retina. Proc Natl Acad Sci USA. (2013) 110:16586–91. 10.1073/pnas.1314575110 24067647 PMC3799310

[16] Khanal S Turnbull PRK Vaghefi E Phillips JR. Repeatability of arterial spin labeling MRI in measuring blood perfusion in the human eye. JMRI. (2019) 49:966–74. 10.1002/jmri.26323 30252997

[17] Cheng H Nair G Walker TA Kim MK Pardue MT Thulé PM. Structural and functional MRI reveals multiple retinal layers. Proc Natl Acad Sci USA. (2006) 103:17525–30. 10.1073/pnas.0605790103 17088544 PMC1859962

[18] Muir ER Rentería RC Duong TQ. Reduced ocular blood flow as an early indicator of diabetic retinopathy in a mouse model of diabetes. Invest Ophthalmol Visual Sci. (2012) 53:6488–94. 10.1167/iovs.12-9758 22915034 PMC4045095

[19] Peng Q Zhang Y Nateras OS van Osch MJ Duong TQ. MRI of blood flow of the human retina. Magn Resonan Med. (2011) 65:1768–75. 10.1002/mrm.22763 21590806 PMC3197718

[20] Agemy SA Scripsema NK Shah CM Chui T Garcia PM Lee JG. Retinal vascular perfusion density mapping using optical coherence tomography angiography in normals and diabetic retinopathy patients. Retina. (2015) 35:2353–63. 10.1097/IAE.0000000000000862 26465617

[21] Hwang TS Gao SS Liu L Lauer AK Bailey ST Flaxel CJ. Automated quantification of capillary nonperfusion using optical coherence tomography angiography in diabetic retinopathy. JAMA Ophthalmol. (2016) 134:367–73. 10.1001/jamaophthalmol.2015.5658 26795548 PMC4978127

[22] Rosenfeld PJ Durbin MK Roisman L Zheng F Miller A Robbins G. ZEISS Angioplex™ spectral domain optical coherence tomography angiography: technical aspects. Dev Ophthalmol. (2016) 56:18–29. 10.1159/000442773 27023249

[23] Duncan RO Sample PA Bowd C Weinreb RN Zangwill LM. Arterial spin labeling fMRI measurements of decreased blood flow in primary visual cortex correlates with decreased visual function in human glaucoma. Vision Res. (2012) 60:51–60. 10.1016/j.visres.2012.03.012 22465941 PMC3340501

[24] Wang Q Chen W Qu X Wang H Wang Y Zhang X. Reduced cerebral blood flow in the visual cortex and its correlation with glaucomatous structural damage to the retina in patients with mild to moderate primary open-angle glaucoma. J Glaucoma. (2018) 27:816–22. 10.1097/IJG.0000000000001017 29952821

[25] Wang H Li S Chen X Wang Y Li J Wang Z. Cerebral blood flow alterations in high myopia: an arterial spin labeling study. Neural Plast. (2020) 2020:6090262. 10.1155/2020/6090262 32399025 PMC7199639

[26] George S Rosenfield M. Blur adaptation and myopia. Optometry Vision Sci. (2004) 81:543–7. 10.1097/00006324-200407000-00016 15252354

[27] Clermont AC Bursell SE. Retinal blood flow in diabetes. Microcirculation. (2007) 14:49–61. 10.1080/10739680601072164 17365661

[28] Al-Sheikh M Akil H Pfau M Sadda SR. Swept-source OCT angiography imaging of the foveal avascular zone and macular capillary network density in diabetic retinopathy. Invest Ophthalmol Vis Sci. (2016) 57:3907–13. 10.1167/iovs.16-19570 27472076

[29] Andersen N Hjortdal J Schielke KC Bek T Grauslund J Laugesen CS. The Danish registry of diabetic retinopathy. Clin Epidemiol. (2016) 8:613–9. 10.2147/CLEP.S99507 27822108 PMC5094648

[30] Pemp B Schmetterer L. Ocular blood flow in diabetes and age-related macular degeneration. Can J Ophthalmol. (2008) 43:295–301. 10.3129/i08-049 18443612

[31] Borogovac A Asllani I. Arterial Spin Labeling (ASL) fMRI: advantages, theoretical constrains, and experimental challenges in neurosciences. Int J Biomed Imaging. (2012) 2012:818456. 10.1155/2012/658101 22966219 PMC3432878

[32] Duong TQ Kim DS Ugurbil K Kim SG. Localized cerebral blood flow response at submillimeter columnar resolution. Proc Natl Acad Sci USA. (2001) 98:10904–9. 10.1073/pnas.191101098 11526212 PMC58572

[33] Calamante F Gadian DG Connelly A. Quantification of perfusion using bolus tracking magnetic resonance imaging in stroke: assumptions, limitations, and potential implications for clinical use. Stroke. (2002) 33:1146–51. 10.1161/01.STR.0000014208.05597.33 11935075

[34] Zhang Y San Emeterio Nateras O Peng Q Rosende CA Duong TQ. Blood flow MRI of the human retina/choroid during rest and isometric exercise. Invest Ophthalmol Vis Sci. (2012) 53:4299–305. 10.1167/iovs.11-9384 22661466 PMC3392013

[35] Chen Y Wang DJ Detre JA. Test-retest reliability of arterial spin labeling with common labeling strategies. J Magn Resonan Imaging. (2011) 33:940–9. 10.1002/jmri.22345 21448961 PMC3069716

[36] Alsop DC Detre JA Golay X Günther M Hendrikse J Hernandez-Garcia L. Recommended implementation of arterial spin-labeled perfusion MRI for clinical applications: a consensus of the ISMRM perfusion study group and the European consortium for ASL in dementia. Magn Resonan Med. (2015) 73:102–16. 10.1002/mrm.25197 24715426 PMC4190138

[37] Johannesen SK Viken JN Vergmann AS Grauslund J. Optical coherence tomography angiography and microvascular changes in diabetic retinopathy: a systematic review. Acta Ophthalmol. (2019) 97:7–14. 10.1111/aos.13859 30238633

[38] Bhanushali D Anegondi N Gadde SG Srinivasan P Chidambara L Yadav NK. Linking retinal microvasculature features with severity of diabetic retinopathy using optical coherence tomography angiography. Invest Ophthalmol Vis Sci. (2016) 57:519–25. 10.1167/iovs.15-18901 27472275

[39] Kim AY Chu Z Shahidzadeh A Wang RK Puliafito CA Kashani AH. Quantifying microvascular density and morphology in diabetic retinopathy using spectral-domain optical coherence tomography angiography. Invest Ophthalmol Vis Sci. (2016) 57:362–70. 10.1167/iovs.15-18904 27409494 PMC4968771

[40] Tang FY Ng DS Lam A Luk F Wong R Chan C. Determinants of quantitative optical coherence tomography angiography metrics in patients with diabetes. Sci Rep. (2017) 7:2575. 10.1038/s41598-017-02767-0 28566760 PMC5451475

[41] Durbin MK An L Shemonski ND Soares M Santos T Lopes M. Quantification of retinal microvascular density in optical coherence tomographic angiography images in diabetic retinopathy. JAMA Ophthalmol. (2017) 135:370–6. 10.1001/jamaophthalmol.2017.0080 28301651 PMC5470403

[42] Mastropasqua R Toto L Mastropasqua A Aloia R De Nicola C Mattei PA. Foveal avascular zone area and parafoveal vessel density measurements in different stages of diabetic retinopathy by optical coherence tomography angiography. Int J Ophthalmol. (2017) 10:1545–51. 10.18240/ijo.2017.10.11 29062774 PMC5638976

[43] Bayraktar Z Alacali N Bayraktar S. Diabetic papillopathy in type II diabetic patients. Retina. (2002) 22:752–8. 10.1097/00006982-200212000-00011 12476102

